# Genetically determined cardiomyopathies at autopsy: the pivotal role of the pathologist in establishing the diagnosis and guiding family screening

**DOI:** 10.1007/s00428-023-03523-8

**Published:** 2023-03-10

**Authors:** Mary N. Sheppard, Allard C. van der Wal, Jytte Banner, Giulia d’Amati, Monica De Gaspari, Rosa De Gouveia, Cira Di Gioia, Carla Giordano, Maiken Kudahl Larsen, Matthew J. Lynch, Joaquin Lucena, Pilar Molina, Sarah Parsons, M. Paz Suarez-Mier, Stefania Rizzo, Simon Kim Suvarna, Wouter P te Rijdt, Gaetano Thiene, Aryan Vink, Joseph Westaby, Katarzyna Michaud, Cristina Basso, on behalf of the Association for European Cardiovascular Pathology (AECVP)

**Affiliations:** 1 CRY Cardiovascular Pathology Unit, Cardiovascular Clinical Academic Group, Molecular and Clinical Sciences Research Institute, St. George’s, University of London, London, UK; 2 Department of Pathology, Amsterdam UMC, University of Amsterdam, Amsterdam, The Netherlands; 3 Section of Forensic Pathology and Clinical Forensic Medicine, Department of Forensic Medicine, Faculty of Health Sciences, University of Copenhagen, Copenhagen, Denmark; 4 Department of Radiological, Oncological and Pathological Sciences, Sapienza University of Rome, Rome, Italy; 5 Cardiovascular Pathology Unit, Department of Cardiac, Thoracic, Vascular Sciences and Public Health, University of Padua, Padua, Italy; 6 Histology and Pathology, Faculty of Life Sciences, University of Madeira & LANA - Clinical and Anatomical Pathology Laboratory, Funchal, Madeira Portugal; 7 Department of Forensic Medicine, Faculty of Health Sciences, University of Aarhus, Aarhus, Denmark; 8 Department of Forensic Medicine, Victorian Institute of Forensic Medicine, Monash University, Melbourne, Australia; 9 Forensic Pathology Service, Institute of Legal Medicine and Forensic Sciences, Seville, Spain; 10 Forensic Pathology Service, Institute of Legal Medicine and Forensic Sciences, Valencia, Spain; 11 Histopathology Service, National Institute of Toxicology and Forensic Sciences, Madrid, Spain; 12 Histopathology Royal Hallamshire Hospital, Sheffield Teaching Hospitals NHS Trust, and University of Sheffield, Sheffield, UK; 13 Department of Clinical Genetics, University Medical Center Rotterdam, Erasmus University Rotterdam, Rotterdam, the Netherlands; 14 Department of Pathology, University Medical Center Utrecht, Utrecht University, Utrecht, Netherlands; 15 University Center of Legal Medicine Lausanne, Lausanne University Hospital and University of Lausanne, Lausanne, Switzerland

**Keywords:** Cardiomyopathies, Autopsy, Sudden cardiac death, Genetic

## Abstract

Cardiomyopathies (CMP) comprise a heterogenous group of diseases affecting primarily the myocardium, either genetic and/or acquired in origin. While many classification systems have been proposed in the clinical setting, there is no internationally agreed pathological consensus concerning the diagnostic approach to inherited CMP at autopsy. A document on autopsy diagnosis of CMP is needed because the complexity of the pathologic backgrounds requires proper insight and expertise. In cases presenting with cardiac hypertrophy and/or dilatation/scarring with normal coronary arteries, a suspicion of inherited CMP must be considered, and a histological examination is essential. Establishing the actual cause of the disease may require a number of tissue-based and/or fluid-based investigations, be it histological, ultrastructural, or molecular. A history of illicit drug use must be looked for. Sudden death is frequently the first manifestation of disease in case of CMP, especially in the young. Also, during routine clinical or forensic autopsies, a suspicion of CMP may arise based on clinical data or pathological findings at autopsy. It is thus a challenge to make a diagnosis of a CMP at autopsy. The pathology report should provide the relevant data and a cardiac diagnosis which can help the family in furthering investigations, including genetic testing in case of genetic forms of CMP. With the explosion in molecular testing and the concept of the molecular autopsy, the pathologist should use strict criteria in the diagnosis of CMP, and helpful for clinical geneticists and cardiologists who advise the family as to the possibility of a genetic disease.

**Supplementary information:**

The online version contains supplementary material available at 10.1007/s00428-023-03523-8.

## Introduction

Cardiomyopathies (CMP) comprise a heterogenous group of diseases affecting primarily the myocardium, with marked variation in clinical presentation, underlying cardiac pathophysiology and etiological background. These backgrounds can be either genetic and/or acquired diseases. As Association for European Cardiovascular Pathology (AECVP), we feel we need a document on autopsy diagnosis of inherited CMP because these entities have undergone major advances in the past 20 years and more precise autopsy diagnosis is essential to move forward with these advances. The complexity of the pathologic backgrounds requires proper insight and expertise, even more since several CMP definitions and diagnosis are still evolving with increased and rapid whole genome testing requiring expert input from many medical professionals. While many classification systems have been proposed in the clinical setting [1–5], and morphologic criteria for the pathological diagnosis of the various CMP are available [6], there is no internationally agreed pathological consensus concerning the diagnostic approach to genetically determined CMP at autopsy. Sudden cardiac death (SCD) is not the only opportunity for a pathologist to establish the true nosology of CMP from cardiac specimens. Cardiac transplantation is also a source of heart specimens. Although usually the diagnosis is already established clinically in this case, sometimes the pathologist can contribute to either alter or refine the diagnosis and the recommendations herein reported apply as well.

In cases presenting with cardiac hypertrophy and/or dilatation/scarring with normal coronary arteries, a suspicion of CMP must be considered, and a histological examination is essential. Establishing the actual cause of the disease may require a number of tissue-based and/or fluid-based investigations, be it histological, ultrastructural, or molecular. A history of illicit drug use must be looked for. The process can also be complicated by the fact that information about disease during life, for example pathogenic genotype, family or drug history may not be available at the time of autopsy. SCD is frequently the first manifestation of disease in case of CMP, especially in the young. Also, during routine clinical or forensic autopsies at any age, a suspicion of CMP may arise based on clinical data or pathological findings at autopsy. It is thus a challenge to make a diagnosis of a potentially genetically determined CMP at autopsy. It is very important that the pathology report provides the relevant data and a cardiac diagnosis which can help the family in furthering investigations, including genetic testing in case of inherited forms of CMP [7–9]. With the explosion in molecular testing and the concept of the molecular autopsy, the pathologist should use strict criteria in the diagnosis of CMP, which should be helpful for clinical geneticists and cardiologists who advise the family as to the possibility of a genetic disease.

## Clinical classifications of CMP

At present, there are three major clinical classifications for diagnosis of CMP, two of them commonly referred to as the American and the European classification, introduced by the American Heart Association (AHA) in 2006 [3] and the European Society of Cardiology (ESC) in 2008 [4], respectively. Later on, the MOGE(S) classification [5] forwarded by World Heart Federation (WHF) in 2013 attempted to provide a phenotype-genotype and etiology based nomenclature. These classifications deal with all the important aspects concerning variations in cardiac pathology and etiological backgrounds, including genetics, but with different approaches. All agree that the term CMP refers to a myocardial disorder in which the heart muscle is structurally and functionally abnormal, in the absence of coronary artery disease, hypertension, valvular disease and congenital heart disease sufficient to cause the observed myocardial abnormality. The AHA defined CMP as a heterogenous group of diseases of the myocardium associated with mechanical and/or electrical dysfunction which usually exhibit inappropriate ventricular hypertrophy and dilatation. Cardiomyopathies were divided into two major groups: primary CMP (which can have a genetic or nongenetic cause) where the pathology is predominantly limited to the heart muscle; and secondary CMP, which have cardiac involvement as part of a large variety of generalized systemic disorders (mostly acquired). The expert panel of the AHA states that CMP are due to a variety of diseases that are frequently genetic, and their classification is primarily based on pathophysiological mechanisms and genetic background [3, 10]. The ESC working group on myocardial and pericardial diseases defined CMP as a myocardial disorder in which the heart muscle is structurally and functionally abnormal in the absence of coronary artery disease, hypertension, valvular disease, and congenital heart disease [4]. Their classification is based on specific morphological and functional phenotypes, i.e., dilated CMP (DCM), hypertrophic CMP (HCM), restrictive CMP (RCM), arrhythmogenic right ventricular CMP (ACM), and provisionally still unclassified CMP. Both documents allude to important distinct features of the various types, but limitations may come up since they mix up entities that are either based on anatomic features (i.e., HCM and DCM) and pathophysiologic features (i.e., RCM and ACM). In these classifications, the same condition can appear clinically in two categories such as cases with dilated and hypertrophic ventricles (mixed phenotype). Subjects with HCM may progress clinically to a dilated phenotype, due to extensive cardiac remodeling during the longstanding course of the disease, and this may be the phenotype seen at autopsy. It should be noted also that in the ESC classification, the so-called channelopathies, a heterogenous group of heritable cardiac diseases characterized by a still-increasing spectrum of ion channel gene mutations with variable but significant risk of arrhythmic SD, are not included because the hearts of these patients usually appear otherwise morphologically normal, clinically as well as at autopsy. The AHA classification, being primarily based on genetics, has included these channelopathies, as primary CMP [3, 10]. For the same reason also conduction defects such as the Lenègre disease (also known as progressive cardiac conduction defect) and sick sinus syndromes are included. The most recent classification from the WHF proposed a nomenclature system addressing five attributes of the CMP, i.e., morpho-functional characteristics (M), organ involvement (O), genetic or familial inheritance pattern (G), and etiological details (E), with the optional addition of functional class/status (S) (i.e., the so-called MOGE(S) classification) [5].

## The pathologist perspective

In the autopsy setting when pathologists suspect the existence of a CMP, either as the cause of death or as an incidental finding, they should follow a protocol that will establish the underlying phenotype of the disease, thus adopting the “morpho-functional” phenotype approach. This phenotypic diagnosis can be based on the pathological investigations alone (the full autopsy including microscopic investigations in the laboratory). However, in other cases, where a final conclusion on the nature of the disease cannot be made or still remains uncertain, the pathology report should include the advice to clinicians, cardiologists, and/or geneticists on how to proceed to a final diagnosis.

The etiology of CMP is diverse and includes genetic, toxic, infectious, or autoimmune causes, for which the pathology findings need to be integrated by further investigations.

If a genetic etiology is suspected, a sample will be provided to test for mutations in genes encoding for sarcomeric, cytoskeletal, nucleoskeletal, mitochondrial, desmosomal, ion channel, and calcium handling proteins which requires the expertise of multidisciplinary cardiogenetic centers (see below). Patho-morphologically, these mutations are most commonly associated with HCM, DCM and ACM phenotypes but overlapping phenotypes do exist [11]. This implies that the most practical approach to a suspected CMP at autopsy will be to apply the ESC classification, which is based on initial grouping of morphological and functional phenotypes, and to follow the proposed flowchart towards sub-classification in familial/genetic forms and nonfamilial diseases with the exclusion of ischemic, valvar, and hypertensive heart disease. Basically, this is the method proposed by Michael J Davies in 1995 and then updated [12–14]. The workup presented in this article aims to follow this approach.

Many CMP patients die of SCD, indicating that pathologists are often in the first line to evaluate a cardiac diagnosis with potential genetic background, which can be crucial for the family members of the deceased [7–9]. It is therefore important to perform an autopsy following the AECVP guidelines, applying the right methodology of dissection, tissue sampling for histology, toxicological and potential genetic analysis [9].

Knowledge of the clinical history and death circumstances (when available) is of crucial importance to raise the possibility of an underlying CMP. A questionnaire distributed to the family of the deceased and/or a team of specifically trained professionals such as nurses could help in obtaining a suitably detailed history from family members.

A previous history of cardiac disease and medications or procedures will be vital. Previous episodes of syncope or pre-syncope, palpitations, or chest pain can be cardiac related. Where there is a possible history of CMP, details concerning this should be obtained and we should proceed with the autopsy with this diagnosis in mind. Also, a family history of SD, sudden infant deaths, unexplained drownings, or accidents will make an underlying genetic cause a possibility.

When a patient dies suddenly with no previous history, extra-cardiac causes of death must be excluded. After a careful examination, a significant number of hearts from patients with SCD can appear structurally normal at autopsy (sudden arrhythmic death syndrome-SADS) [15–26]. Such a finding might underlie the heterogenic group of (familial) ion channel mutation related diseases which include the long QT, short QT, and Brugada syndromes and polymorphic catecholaminergic ventricular tachycardia. As mentioned above, they are not included in the ESC classification of CMP and cannot be diagnosed at autopsy due to lack of structural changes. Therefore, the identification of a structurally normal heart in case of arrhythmic SCD is of equal importance as it does not exclude the presence of a genetically determined and potentially hereditary cardiac disease and a further evaluation with a multidisciplinary team approach is needed. Clear-cut criteria to define a normal heart are crucial [9, 16, 27–30]. Obviously, a detailed drug history is important and use of cocaine, steroids and methamphetamine sought as these can cause both hypertrophy and dilatation of the heart. Cases with uncertain pathology findings and drug concentration below the lethal threshold should be carefully interpreted [15, 17, 18].

The finding of a pathogenic or probably pathogenic mutation in genes associated with CMP in cases with normal or near normal heart pathology raises the possibility of a SCD in an electrically unstable “early phase” of the disease, without enough structural disease to receive a postmortem diagnosis of CMP. Since still a large proportion of SADS victims exhibits no mutation in ion channel genes, these cases must prompt a comprehensive cardiological family screening looking for channelopathies as well as for CMP features in at risk relatives and an appropriate cascade genetic study should be offered if the CMP-related mutation is considered pathogenic/probably pathogenic.

## Autopsy in suspected CMP

The pathologist should examine the heart according to a protocol already set out in guidelines on the investigation of SCD [9, 31] and also on cardiac hypertrophy [32]. Once diseases of coronary arteries, great vessels and valves are excluded, then the pathologist must suspect that the morphological cause could be within the myocardium. No significant coronary artery disease and hypertension are very common especially in older patients and should not be given as the cause of death without performing cardiac histology. Histological sampling of both ventricles including the interventricular septum (IVS) must be done, usually at mid ventricular level as previously reported.

The pathologist should be aware that a cardiac diagnosis made during life may not necessarily be in agreement with the autopsy findings in the heart. When another cause of death is found, but the heart looks potentially “cardiomyopathic,” tissue sampling of the heart is advised as indicated and material for genetic testing should be taken (if possible, blood in EDTA tube or a small piece of fresh spleen). In some medicolegal settings, this may be discouraged in view of costs but we believe the pathologist has a duty of care to inform the coroner/ district attorney and family of this possibility.

Due to widespread occurrence of coronary artery disease in the population, co-occurrence of obstructive atherosclerotic plaques in the coronary arteries of hearts with CMP is not uncommon, particularly in the older age groups. Pathologists should be aware of possible co-existence of highly prevalent acquired disease in these patients in order to avoid misdiagnosis or under-diagnosis. The same is also true for hypertension, especially in males over 40 years [9]

As recommended in the guidelines for autopsy investigation of SCD, in the absence of local expertise in cardiovascular pathology, the best practice is that the entire heart is retained and sent to a specialized center for an expert opinion [9]. The referring pathologist should complete the initial steps, including making a transverse apical section of the heart and emptying the heart of blood before referral. If the heart cannot be retained, it is essential that extensive photographic documentation is made, indicating where individual blocks are taken. At least one transverse section of the heart including the LV and RV should be retained for further examination.

## Macroscopic and histologic “red flags” with diagnostic pitfalls

**Fig. 1:**
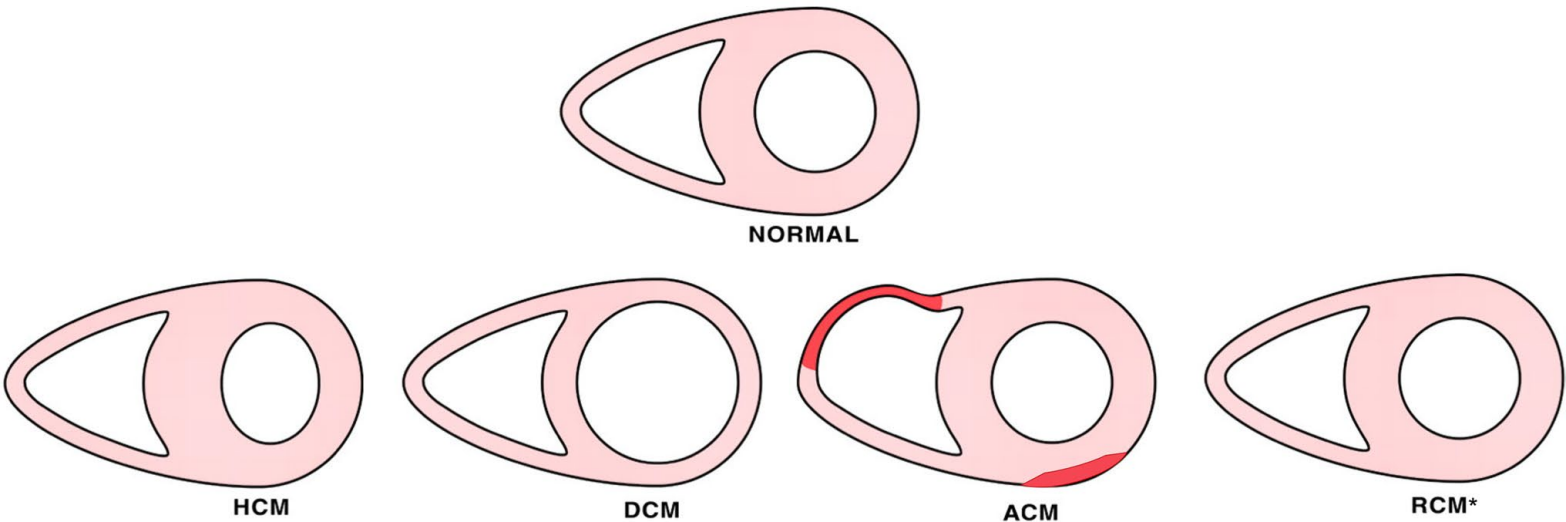
Diagrammatic representation of the gross phenotype of normal heart and primary inherited cardiomyopathies at short axis cut. Normal heart, hypertrophic cardiomyopathy (HCM), dilated cardiomyopathy (DCM), arrhythmogenic cardiomyopathy (ACM), and restrictive cardiomyopathy (RCM). Note that in primary RCM, at cross section, the phenotype is usually not distinct from normal heart, and atrial dilatation in the absence valvular and endocardial diseases is the red flag at gross examination

The following paragraphs will address the more distinctive gross and histologic features of the four main genetically determined CMP phenotypes (see also Table 1 and Fig. 1). For definitions of terms, please refer to Table 1 from Basso et al. [32].

**Table 1:** Main gross and histologic features of primary inherited cardiomyopathies

	HCM	ACM	DCM	RCM
Gross features				
Heart size/weight	Increased/normal	Normal/increased	Increased/normal	Normal
Wall thickness	Increased/normal/decreased	Normal/decreased/increased	Decreased/normal	Normal
RV dilatation	No	No/yes	Yes	No
LV dilatation	No/yes	No/yes	Yes	No
Atria	Normal/dilated LA	Normal/dilated RA	Normal/dilated LA	Very dilated RA-LA
Valve abnormalities	No/MV anterior leaflet thickening	No/TV anulus dilatation	MV anulus dilatation and PM displacement	No
Coronary arteries	Possible myocardial bridge	No disease-related abnormalities	No disease-related abnormalities	No disease-related abnormalities
Myocardial hypertrophy	Usually concentric, various patterns	No	Usually eccentric	No
Myocardial scarring LV	No/yes, multifocal	No/yes, subepicardium-midmural-transmural	No/yes, midmural confluent	No
Myocardial scarring RV	No	Yes	No	No
Myocardial fat in LV	No	No/yes	No	No
Myocardial fat in RV	No	Yes/no	No	No
Aneurysms	No/yes LV apical	No/yes, usually RV multiple locations	No	No
Hypertrabeculation	No/yes LV	No/Yes LV RV	No/Yes, LV RV	No
Endocardium	Normal/impact lesion LVOT	Normal/mural thrombosis	Mural thrombosis/organized/fibroelastosis	Normal
Pericardium	Normal	Normal	Normal	Normal
Phenocopies/differential diagnosis	Hypertension, storage, metabolic, amyloidosis, aortic stenosis, aortic coarctation, elderly basal septal hypertrophy, anabolic drug abuse, idiopathic LVH, chronic IHD	Fatty heart, chronic myocarditis, sarcoidosis, muscular dystrophy, Chagas disease, chronic IHD, DCM	Muscular dystrophy, acute myocarditis, chronic myocarditis, sarcoidosis, end-stage HCM, ACM L, chronic IHD, toxic, alcohol, drugs	Loeffler endocarditis EMF, amyloidosis, sarcoidosis hemochromatosis
Histologic features				
Myocyte hypertrophy	Yes	No/yes	Yes	No
Myocyte disarray	Yes	No	No	Yes
Myocyte degeneration	Yes	Yes	Yes	No
Interstitial fibrosis	Yes	Yes	Yes	Yes
Replacement fibrosis	No/Yes (midmural, multifocal subendocardial)	Yes (subepicardium-midmural-transmural)	No/Yes (midmural confluent)	No
Endocardial fibrosis	No/yes	No/yes	Yes	No
Inflammation	No	Yes/no	Yes/no	No
Fibro-fatty tissue RV/LV	No	Yes (subepicardium- midmural-transmural)	No	No
Obstructive SVD	Yes/no	No	No	No

ACM, arrhythmogenic cardiomyopathy; ACM-L, arrhythmogenic cardiomyopathy left variant; DCM; dilated cardiomyopathy; EMF; endomyocardial fibrosis; HCM, hypertrophic cardiomyopathy; IHD, ischemic heart disease; LA, left atrium; LV, left ventricle; LVH, left ventricular hypertrophy; LVOT; left ventricular outflow tract; MV, mitral valve; PM, papillary muscles; RCM, restrictive cardiomyopathy; RA, right atrium; RV, right ventricle; SVD; small vessel disease; TV, tricuspid valve

### Hypertrophic cardiomyopathy

At autopsy, there is usually a clear and appropriate cause for left ventricular (LV) hypertrophy such as aortic stenosis or hypertension [32]. When there is no clear cause then HCM should be considered and the diagnosis is often made first at autopsy [19, 32]. Obviously, if the pathologist is already aware of the diagnosis, confirmation should be obtained at autopsy.

According to ESC guidelines on HCM, the definition of HCM applies to children and adults and makes no a priori assumptions about etiology or myocardial pathology [33].

HCM occurs in approximately 1:500 of the general population [11, 33]. It is the most common inherited monogenic cardiac condition. Most HCM have an autosomal dominant pattern of inheritance caused by mutations in genes that encode different proteins of the cardiac sarcomere. Currently, there are over 500 known mutations in 11 genes (beta-myosin heavy chain; cardiac myosin-binding protein C; cardiac troponin-T; troponin I; alpha-tropomyosin; essential and regulatory myosin light chains; actin; alpha-myosin heavy chain; titin; and muscle LIM protein). In most patients, HCM is caused by the first three genes while the other genes each account for only a small fraction. The diagnostic yield of genetic testing is 60–70% [11, 33, 34].

Up to 10% of adult cases are due to other genetic/nongenetic disorders (phenocopies), such as metabolic disorders (Anderson-Fabry, Danon, and Pompe diseases), mitochondrial CMP, neuromuscular disease (Friedreich’s ataxia), malformation syndromes (Noonan, LEOPARD, and Costello syndromes), infiltrative disease (amyloidosis), and endocrine disorders (infants of mothers with diabetes, phaeochromocytoma, and acromegaly). Clinical history, metabolic studies, autopsy findings in other organs, histology, and genetic testing are essential in the diagnosis of these entities [32, 34].

#### Macroscopic changes in sarcomeric HCM (Fig. 2)

**Fig. 2:**
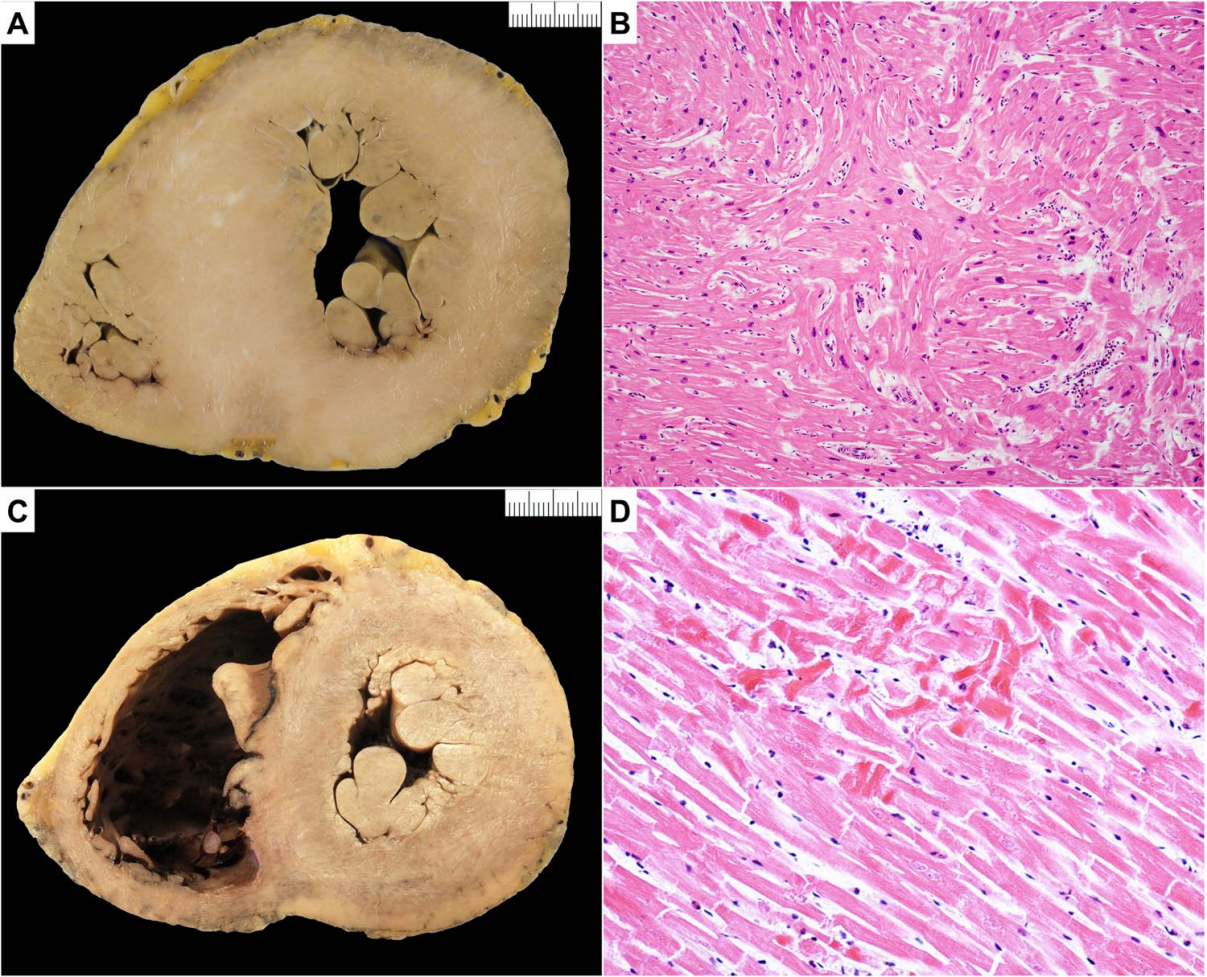
Hypertrophic cardiomyopathy (HCM) and pitfall (postmortem hypercontraction). **A** Macroscopic short axis cut with circumferential hypertrophy of the left ventricle. There is also prominent septal hypertrophy with anteroseptal irregular scarring. Note also prominent papillary muscles and trabeculae in the left ventricle. **B** Microscopic section shows individual myocyte and bundle disarray with hyperchromatic nuclei. There is an increase in interstitial collagen, findings in keeping with HCM (hematoxylin and eosin stain). **C** Macroscopic short axis cut with circumferential increase in thickening of the left ventricle due to hypercontraction. There is no scarring or prominent septal hypertrophy. Note also the prominent papillary muscles and trabeculae in left ventricle. **D** Microscopic section shows myocyte hypercontraction with contraction bands in otherwise normal myocytes. There is no increase in interstitial collagen (hematoxylin and eosin stain)

- *Heart size*. The heart is usually enlarged with increase in transverse and longitudinal dimensions and may have obvious bulging of the LV externally.
- *Heart weight* is often but not necessarily increased. See normal values for age and body weight [32].
- *Wall thickness*. IVS and LV thickness are usually increased (see referral normal values) ranging from 15 to > 50 mm. Normal weight and wall thickness are reported in up to 30% of cases [35] and particularly in patients with troponin T and some mutations of the myosin gene. Right ventricular (RV) wall thickness can be increased > 5 mm.
- *Ventricular cavity size* is usually reduced. LV cavity enlargement can be observed in advanced end stage forms.
- *Atria*. Left atrial (LA) cavity can be increased.
- *Valves*. The anterior leaflet of the mitral valve can be thickened due to friction lesion. There can be thickening with ballooning and elongation of both anterior and posterior mitral leaflet with elongation also of the attached cords [36]. Papillary muscle hypertrophy and displacement may occur with insertion of the anterolateral papillary muscle directly into the mid-anterior mitral leaflet without intervening cords.
- *Coronary arteries*. Myocardial bridge, i.e., a deep intramyocardial course of the left anterior descending coronary artery, is reported in one fourth of HCM cases but is only significant if associated with ischemia or fibrosis in the anteroseptal wall of the LV [37].
- *Myocardium*. The majority of patients with sarcomeric protein gene mutations have an asymmetrical pattern of hypertrophy. Basal anterior IVS and anterior LV free wall are the most common location. Any pattern and distribution of LV wall thickening, including apical forms, as well as biventricular involvement can be observed.

Bundle disarrangement/whorling can be seen macroscopically in areas of hypertrophy. Irregular pale scars can be seen usually in the IVS and the free wall, usually in the most hypertrophied regions. When transmural fibrosis is present, it could lead to wall thinning which can mimic DCM or chronic ischemic heart disease. Hypertrabeculation with increased thickness of the trabecular layer can occur with deep crypt formation. Aneurysms are rare and can occur usually in the apical forms of the disease.

- *Endocardium*. Impact/friction lesion is a patch of pale fibrotic endocardial thickening due to the impact of the anterior mitral valve leaflet on the upper IVS. This is associated with basal IVS hypertrophy and LV outflow tract obstruction but may not be present in all cases.
- *Pericardium*. No disease-related abnormalities.

#### Microscopic changes in sarcomeric HCM

Both hypertrophied and no hypertrophied areas should be sampled. The septal areas and outer free wall are the best areas to look for myocyte hypertrophy and disarray. Myocyte disarray is the most important diagnostic histological feature of HCM. One should avoid interpreting histology from RV as there is prominent trabeculation in this chamber and myocyte disarray is a normal feature around these trabecular regions. The same is for the anterior and posterior septal walls and their junction with the free wall and the subendocardium around the trabeculae as myocyte disarray and increased collagen will be seen here normally. Look also for myocyte hypertrophy with disorganized cellular architecture, myocardial scarring and expanded interstitial collagen.

- *Cardiac myocytes*. Hypertrophy is always present. The myocytes show bizarre shapes, nuclear pleomorphism and hyperchromasia.

Myocyte disarray is always present. The hypertrophied cardiac myocytes show multiple intercellular connections, often arranged in chaotic alignment at oblique and perpendicular angles to each other or around capillaries as whorls of myocytes. Individual cellular and bundle disarray can be widely distributed, occupying substantial portions of the IVS and LV free wall [35, 38–40]. Look in areas away from significant replacement fibrosis to search for disarray. It is also common to find sections of myocardium which do not show myocyte hypertrophy or disarray even in hypertrophied areas macroscopically.

- *Fibrosis*. General increase in interstitial fibrosis is always present. Foci of loose connective tissue in the center of whorls of myocytes are classical findings [41, 42]. Replacement-type fibrosis is common and is found in the subendocardium as well as scattered large confluent areas in the IVS and free wall of the LV. This fibrosis is due to ischemic myocardial injury. Acute infarction or healing granulation tissue is also seen but is rare.
- *Inflammatory cells*. No disease-related inflammatory changes are reported.
- *Intramural small vessels*. Abnormal intramural coronary arteries, usually labelled as small vessel disease, characterized by thickened walls with increased intimal and medial collagen and narrowed lumen can be observed especially in areas with replacement-type fibrosis but this feature is not present in all cases [43–45].

#### Diagnostic challenges

##### *Myocyte disarray*

When the myocyte disarray is very focal, we would advise to repeat sampling of the LV including IVS and free wall which often will show more myocyte disarray to come to a definitive diagnosis. There will always be cases where the myocyte disarray is so subtle that a definite diagnosis cannot be made histologically even with extensive cardiac sampling at autopsy. In these cases, it is best to leave the diagnosis as inconclusive and possibility of HCM must be still included, and advise family screening with cascade genetic testing to solve the dilemma. Myocardial disarray in the RV is not diagnostic.

Several systemic diseases (sometimes with isolated cardiac involvement) may present as HCM phenocopies (such as glycogen storage diseases, Fabry disease, and mitochondrial diseases). The histological findings of abnormal cardiomyocyte vacuolization and/or marked inflammation may raise this suspicion, especially if personal or family history include red flags of those entities, and prompt to ask for other diagnostics tests [46]. The presence of accessory pathways should raise the suspicion of PRKAG2 and LAMP2-related HCM (see Basso et al. for more details) [32].

In desmin myopathy, the disease is mainly expressed in skeletal muscle, but cardiac involvement does occur and rarely cardiac involvement is predominant. Myocytes contain eosinophilic conglomerates of fibrillary material staining by immunohistochemistry for desmin. Desmin accumulation may be difficult to be recognized with the light microscopy even at endomyocardial biopsy and the gold standard for the diagnosis of desmin accumulation remains the ultrastructural study [47].

In children, HCM phenotype with disarray histologically is often associated with congenital syndromes, inherited metabolic disorders and neuromuscular diseases, unrelated to sarcomere gene mutations.

In patients with systemic hypertension, extreme circumferential LV hypertrophy can be present. Histologically there is marked myocyte hypertrophy with separation of myocytes and expansion of the interstitium [48].

Hypercontraction of the LV may occur in SD with decreased chamber size, but there will be no increase in heart weight and histologically there will hypercontracted myocytes but not myocyte disarray or fibrosis.

### Arrhythmogenic cardiomyopathy (Fig. 3)

**Fig. 3:**
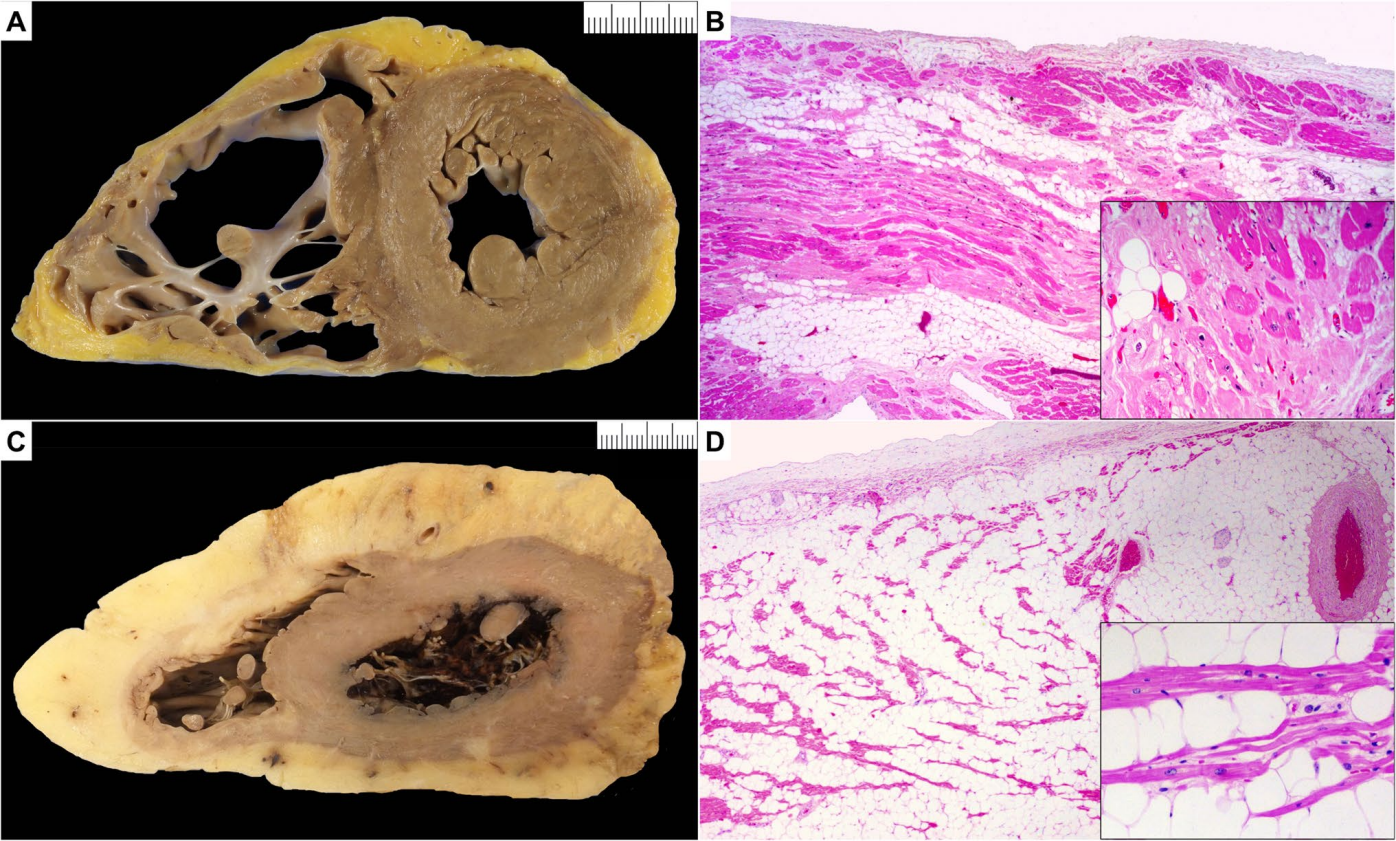
Arrhythmogenic cardiomyopathy (ACM) and pitfall (fatty heart). **A** Macroscopic short axis cut with fat infiltration from epicardial surface of the anterior, lateral, and posterior wall of the right ventricle with fat infiltration also of the lateral left ventricle wall. **B** Microscopic section shows fat admixed with collagen. There is individual myocyte and bundle degeneration (insert) in keeping with ACM (hematoxylin and eosin stain). **C** Macroscopic short axis cut with circumferential increase in epicardial fat surrounding both right and left ventricle. **D** Microscopic section shows mature fat admixed with myocyte bundles. There is no myocyte degeneration or increase in collagen (hematoxylin and eosin stain)

The ACM phenotype is characterized by fibro-fatty or fibrous replacement of the subepicardial myocardium. Fibro-fatty infiltration with a subepicardial or mid-mural location in both RV and LV are therefore the most common reasons to suspect ACM at gross examination [49–53].

The process starts in the outer layers and extends toward the endocardium and can become transmural. Three variants are described depending on the ventricular chamber mostly affected: classical RV (ACM R), biventricular (ACM B), and LV dominant (ACM L) variant [54, 55]. The normal RV has prominent epicardial fat without fibrosis in the anterior and lateral wall while fat plus fibrosis in the posterobasal RV wall points to ACM R. In ACM L, epicardial fat with linear scarring is more pronounced in the posterobasal wall.

ACM occurs in approximately 1:2000–1:5000 of the general population [56]. It is a well-known cause of SCD in the young and athletes often as the first presentation of the disease. ACM phenotype is reported in childhood only in recessive variants such as Carvajal and Naxos syndromes.

#### Genetic background

It is mainly an inherited CMP, mostly with an autosomal dominant pattern of inheritance caused by pathogenic mutations in genes encoding structural proteins of intercellular junctions, mostly desmosomes, such as plakophilin (*PKP2*), desmoplakin (*DSP*), desmoglein (*DSG2*) and desmocollin (*DSC2*), and rarely (< 1%) for the “area composita,” such as α-T-catenin (*CTNNA3*) and N-cadherin (*CDH2*). ACM-causing mutations have also been found in nondesmosomal genes such as phospholamban (*PLN*), filamin-C (*FLNC*), desmin (*DES*), titin (*TTN*), and lamin A/C (*LMNA*), which are associated with other CMP, such as DCM and neuromuscular CMPs, and may lead to overlapping phenotypes. Causal mutations in nondesmosomal genes, such as transmembrane protein 43 (*TMEM43*), SCN5A, and transforming growth factor beta-3 (*TGFß-3*) genes among the others, have been rarely identified. The diagnostic yield of genetic testing in ACM is approximately 50% [56].

While the ACM-R is often linked to desmosomal gene mutations, ACM-L is more problematic as the phenotype of fibro-fatty replacement with prevalent fibrous tissue in the LV may also be the result of healed infarction, myocarditis or toxic agents so care is needed with family follow-up and genetic testing to help in the possible genetic etiology.

#### Macroscopic changes

- *Heart size*. The heart can be normal in size or enlarged with increase in transverse and longitudinal size. At external view, yellow or whitish appearance can be found on the epicardial surface. Aneurysms, at autopsy usually present as local thinning of the ventricular wall, are typically seen in the classical ACM R variant, in the so-called triangle of dysplasia (RV inflow, outflow, and apex).
- *Heart weight*. The heart weight can be normal or increased. See normal values for age and body weight [32].
- *Wall thickness*. LV and IVS thickness can be either normal or decreased in ACM B or ACM L. RV wall thickness either normal or decreased in classical ACM R; pseudo-hypertrophy due to increase in epicardial fibro-fatty tissue is also described.
- *Ventricular cavity size* can be either normal or enlarged.
- *Valves*. ACM R with RV chamber dilatation and annulus enlargement, with or without sub-tricuspid RV inferior wall thinning, can underly tricuspid valve incompetence.
- *Coronary arteries*. No disease-related abnormalities.
- *Atria*. *Right atrial* (RA) enlargement is described in advanced ACM R with appendage thrombi.
- *Myocardium*. A normal appearance of the heart at macroscopic examination does not exclude ACM. Multiple cross sections of the hearts are required to rule out ACM at autopsy due to focal disease distribution. The classical ACM R is often characterized by transmural fibro-fatty replacement with residual myocardium only in the trabeculae, accounting for free wall thinning, translucency, and aneurysm formation. There can be compensatory hypertrophy of the residual myocardium in the trabeculae giving impression of hyper-trabeculation. The ACM L variant is characterized by preserved RV wall thickness and either normal size/increased thickness of LV. There can be enlarged LV cavity with thinned free wall particularly the posterobasal wall; compensatory hypertrophy of the trabeculae is reported in advanced forms; aneurysms are exceptional. The replacing fibrous or fibro-fatty tissue is usually confined to the outer layers (epicardial/midmural). Sometimes the abnormal subepicardial band is so thin that only a fine band-like depression is seen at the macroscopic examination and can easily be missed.

The ACM B variants are characterized by both ACM R and ACM L features. The replacing tissue is mostly fibro-fatty tissue in the RV and fibrous tissue in the LV. There can be prominent epicardial fat particularly in the RV anterior and lateral wall in older often female obese patients, so histology is essential for differential diagnosis. Prominent epicardial fat especially infiltrating the myocardium is very unusual in the LV, so is more suspicious of the diagnosis of ACM L but again histology is essential. The IVS is rarely involved and if so, it is mostly affected at its right side. Circumferential LV involvement suggests FLNC or DSP mutations as first genotype options [57, 58].

- *Endocardium*. Fibrous thickening with white appearance is described in advanced stages, with or without mural thrombosis.
- *Pericardium*. No disease-related abnormalities.

#### Microscopic changes

Both normal and abnormal areas should be sampled. Look for replacing fibrous tissue and fibro-fatty tissue and cardiac myocyte degenerative features.

- *Cardiac myocytes*. Histological features can vary from mild to severe degenerative changes with nuclear abnormalities and cytoplasmic vacuolation to cell death.
- *Fibrosis*. ACM is characterized by replacement fibrosis which starts in the epicardium or mid-mural layers. Both replacement and interstitial fibrosis can be observed. In the RV it is admixed with fat. In the LV, it may be mainly interstitial and replacement fibrosis with little fat. Endocardial fibrosis can be present. Fibro-fatty tissue replacement in the RV is always present, at the intersection of the free wall with epicardial fat; in the LV, it can be present but usually there is more fibrous tissue. Isolated fatty tissue without fibrosis is not a diagnostic feature of ACM [59, 60].
- *Inflammatory cells*. Focal sparse lympho-monocyte infiltrates can be present similarly to DCM. Sometimes diffuse inflammatory infiltrates preferentially affecting the LV in the subepicardial and/or mid-mural layers are observed which makes difficult or impossible the differential diagnosis between ACM (acute/hot phase) and myocarditis [49, 61]. Granulation tissue and loose connective tissue have been also described in subacute stages of disease evolution.
- *Intramural small vessels*. No disease-related abnormalities.

#### Diagnostic challenges and mimics

An increased amount of epicardial fat (*adipositas cordis*) and isolated fatty infiltration in the RV are different entities and should not be confused with ACM. They can be observed in obese people, people with metabolic diseases, elderly or even normal people, with or without increased epicardial fat, particularly in the anterior and lateral RV free walls [59, 60]. Fat also accompanies the intramural vessels in both ventricles. A certain amount of fatty tissue is always present within the myocardium of the RV free wall so that strands of myocardial fibers are separated by fatty tissue, without cardiac myocytes abnormalities and replacement-type fibrosis. The boundary between the myocardium and the outer epicardial fat is usually distinct. Focal areas of endocardial fat with no fibrosis are also a nonspecific finding that should not led to a diagnosis of ACM.

Myocardial fibrosis, with a nonischemic distribution in the LV free wall, either patchy or confluent in the mid-mural or sub-epicardial layers, can be due to a previous myocarditis, toxic insult or infarction. A descriptive pathology report of this to include all possibilities should be provided and advise molecular autopsy with cardiogenetic screening of first degree family members [51, 62].

Differential diagnosis of ACM also includes DCM, isolated cardiac sarcoidosis, Chagas diseases, previous myocardial infarction, muscular dystrophies, and chronic effects of drug toxicity. Abnormal pulmonary venous drainages or other congenital heart defect with RV overload and dilatation should be excluded.

Histological features and/or clinical history and other cardiac or extracardiac findings are of help in differential diagnosis.

### Dilated cardiomyopathy (Fig. 4)

**Fig. 4:**
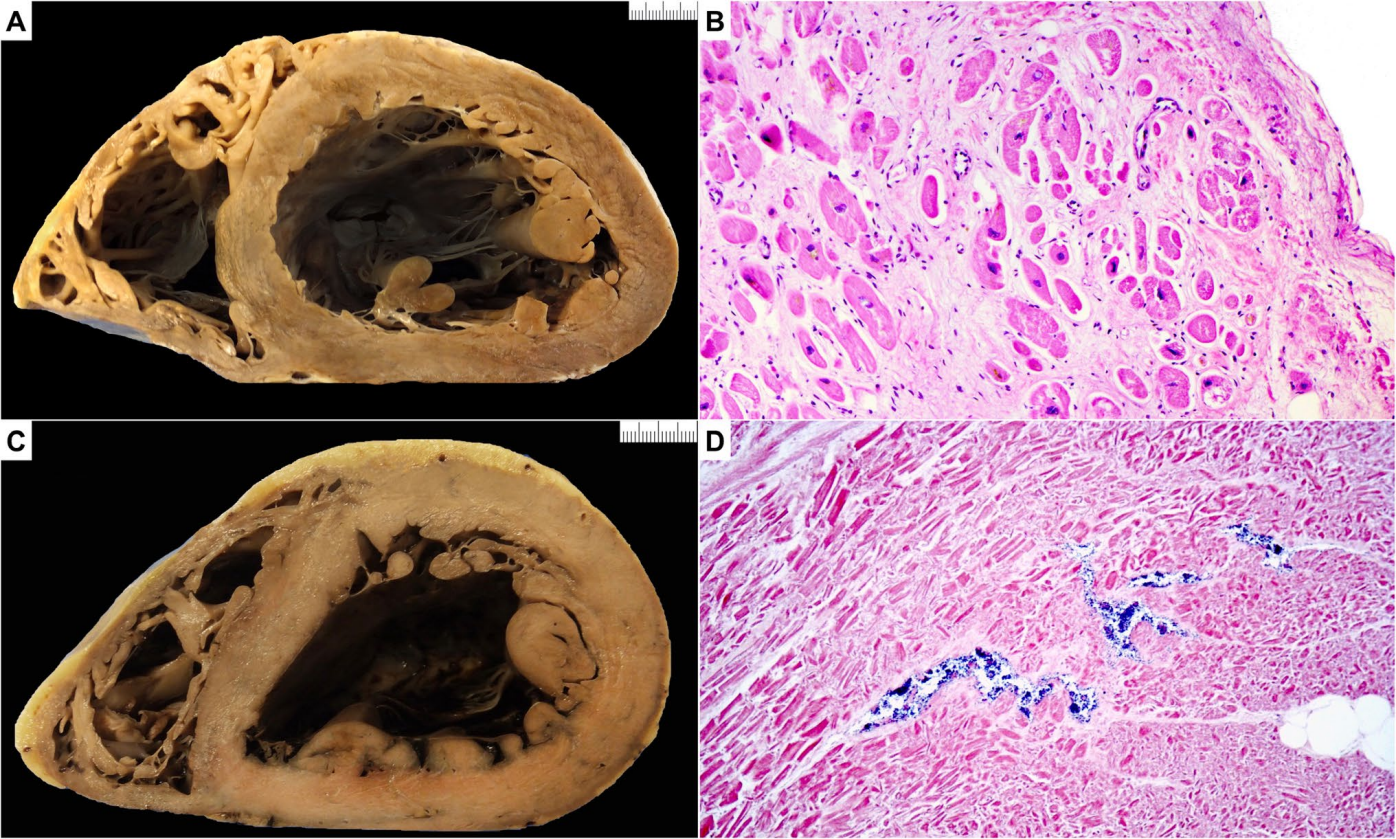
Dilated cardiomyopathy (DCM) and pitfall (postmortem putrefaction). **A** Macroscopic short axis cut with dilated left ventricle chamber and thinning of the left ventricular free wall. **B** Microscopic section shows interstitial and replacement collagen admixed with hypertrophied and degenerate myocytes in keeping with DCM (hematoxylin and eosin stain). **C** Macroscopic short axis cut with dilated left ventricle chamber and thinning of the left ventricular free wall. Note also the dark discoloration of both right and left ventricle. **D** Microscopic section shows hazy myocytes with separation of individual myocytes and myocyte bundles. There is bacterial proliferation within capillaries. Gas bubble formation is also noted (hematoxylin and eosin stain)

It is the most common CMP and includes genetic and nongenetic forms [63, 64]. Its prevalence has been recently estimated in the range of 1:250, and the percentage of genetically determined forms ranges from 30 to 50%. The presence of cardiomegaly with increased cardiac mass and LV or biventricular dilatation with decreased mass/volume ratio, in the absence of coronary artery and valve diseases and hypertension, should led to the suspicion of DCM.

Familial DCM accounts for 30–50% of DCM. In patients with familial DCM, approximately 40–50% has an identifiable genetic cause [64]. The genetic background of DCM is wide and complex with pathogenic mutations in more than 50 genes encoding for cytoskeleton, sarcomeric proteins, sarcolemma, nuclear envelope, ion channels, and intercellular junctions. Although mutations in titin remain the most common identifiable cause, there is growing evidence for mutations in lamin A, desmosomal genes, and filamin C gene underlying an arrhythmogenic form of DCM. X-linked, autosomal recessive, and mitochondrial inheritance of DCM are less common. Phospholamban (PLN) CMP has clinical and histological characteristics of both DCM and ACM.

Acquired causes of DCM include alcohol, pregnancy, post-myocarditis, hemochromatosis, chronic anemia, anthracycline medications, sarcoidosis, stimulant drugs (e.g., cocaine), etc. Idiopathic DCM is eventually a diagnosis of exclusion. The recent demonstration that up to 20% of DCM patients with an established acquired risk factor or a nonfamilial disease still carries a pathogenic gene variant suggests a broader role for genetic testing in DCM [65].

From the pathological viewpoint, there is no evidence that particular patterns or amount of fibrosis and cardiac myocyte changes will favor either acquired or genetic cause. A diagnosis of DCM should always trigger familial investigation and genetic testing for an etiologic diagnosis.

#### Macroscopic changes

- *Heart size.* The heart is enlarged with increased transverse size [12].
- *Heart*. The heart weight is usually increased. See normal values for age and body weight [32].
- *Wall thickness*. IVS and LV wall thickness are usually normal values or even decreased due to wall thinning (see referral normal values) [32]. RV wall thickness is normal or decreased.
- *Ventricular cavity size*. LV cavity enlargement is typically observed, internal diameter can help for qualitative assessment (eccentric hypertrophy) [32]. RV cavity can be also enlarged. If chamber dilatation is the only finding especially with delayed autopsy, one should look for other macroscopic and microscopic findings to support the suspicion of DCM.
- *Valves*. DCM with chamber dilatation and mitro-tricuspid annulus enlargement could explain the in vivo finding of functional regurgitation. Mild increase in leaflet thickening can be observed.
- *Coronary arteries*. No disease-related abnormalities.
- *Atria*. RA and LA cavity can be dilated. Endocardial thrombosis can be present, particularly in the appendages.
- *Myocardium*. Fibrous scar may be present, of variable size and distribution typically with a nonischemic pattern. Sometimes distinctive patterns, such as midwall fibrosis particularly in the IVS, are visible. Hypertrabeculation can occur in advanced forms with wall thinning and prominent cavity dilatation.
- *Endocardium*. Mural thrombosis is frequent due to blood stagnation in the poorly contracting LV. With time organizing thrombosis can lead to whitish appearance of the endocardium with fibrous thickening. Endocardial fibrosis can also be observed in the absence of previous thrombus. Fibroelastosis of the LV endocardium is common in the first years of life, usually associated with viral infection or congenital anomalies [66].
- *Pericardium*. Usually no disease-related abnormalities. The presence of pericardial adhesion could indicate that DCM is a result of previous myo-pericarditis.

#### Microscopic changes

The histologic abnormalities are nonspecific.

- *Cardiac myocytes*. Histological features can vary from normal to slight to marked degenerative changes with increased cell diameter, nuclear abnormalities and cytoplasmic attenuation with perinuclear halo. Hyperchromatic and asymmetric nuclei are often observed.
- *Fibrosis*. Interstitial fibrosis of variable degree is present; replacement-type fibrosis can be present with either patchy or confluent non-ischemic distribution.
- *Inflammatory cells*. Focal sparse lympho-monocyte infiltrates can be present.
- *Intramural small vessel*. No disease-related abnormalities.

#### Diagnostic challenges and mimics

Differential diagnosis of DCM includes myocarditis, isolated cardiac sarcoidosis, end-stage HCM, ACM, muscular dystrophies, and chronic ischemic heart disease with LV remodeling. Specific histological features and/or clinical history and other cardiac or extracardiac findings are of help in differential diagnosis. Pathologists should take in mind that chamber dilatation can be observed as an artefact of postmortem autolytic changes as already stated. Therefore, if chamber dilatation is the only finding, one should look for other macroscopic and microscopic findings that support the diagnosis of DCM.

### Restrictive cardiomyopathy

Primary restrictive CMP (RCM) is a poorly recognized entity characterized by nondilated, nonhypertrophied ventricles with dilated atria. The heart may be more rigid with cutting. In comparison with other forms of CMP, RCM is uncommon and a rare a cause of SCD. The pathology diagnosis is challenging in the absence of clinical findings which are essential to support a certain diagnosis of RCM [67].

Many of the RCM probands had pathologic mutations in either beta-myosin heavy chain (*MYH7*) or the cardiac troponin I gene (*TNNI3*) [68]. Mutations in other sarcomeric genes including troponin T (*TTNT2*), myosin-binding protein C (*MYBPC3*), myosin light chains (*MYL 2* and *3*), and alpha cardiac actin (*ACTC*) have also been described. RCM can coexist with HCM in the same family. Most sarcomeric RCM mutations appear to be de novo and associated with a severe disease expression and an early onset.

Nonsarcomeric mutations have also been identified in RCM and include mutations in myopalladin (*MYPN*), titin (*TTN*), and filamin-C (FLNC) [68].

Genetic causes cannot be established from macroscopic or microscopic features and family investigation with appropriate genetic testing is essential.

Pathologists should be aware that there are several other causes of RCM, including infiltrative diseases (amyloidosis in which the amyloid may be mistaken for fibrosis), storage diseases, mitochondrial disease and a variety of systemic diseases such as hemochromatosis. In these cases, there may be overlap with the other CMP phenotypes such as HCM.

Loeffler endocarditis and endomyocardial fibrosis can also present with a restrictive pattern.

Histological features and/or clinical history and other cardiac or extracardiac findings are essential in making these diagnoses. Interstitial fibrosis with or without myocyte disarray, without evidence of storage or infiltrative disease, are highly suggestive histologic features of primary RCM.

#### Macroscopic changes

- *Heart size*. The heart is usually normal in size with normal transverse and longitudinal size.
- *Heart weight* usually normal. See normal values for age and body weight [32].
- *Wall thickness*. IVS, LV, and RV wall thickness usually normal values (see referral normal values).
- *Ventricular cavity size*. Normal.
- *Valves*. No disease-related abnormalities.
- *Coronary arteries*. No disease-related abnormalities.
- *Atria*. RA and LA cavity can be dilated. Endocardial thrombosis can be present, particularly in the appendages [69].
- *Myocardium*. No disease-related abnormalities.
- *Endocardium*. No disease-related abnormalities.
- *Pericardium*. No disease-related abnormalities.

#### Microscopic changes

- *Cardiac myocytes*. They are usually of normal size and morphology or may show degenerative changes. Myocyte disarray can be observed as part of HCM spectrum [69, 70].
- *Fibrosis*. Interstitial fibrosis is present in all cases. Replacement-type fibrosis can be present.
- *Inflammatory cells*. No disease-related changes are reported.
- *Intramural small vessel*. No disease-related abnormalities.

### Uncertain categories/findings

The entity reported as noncompaction CMP, which is also known as LV noncompaction (LVNC), is characterized by excessive trabeculations of the LV and deep intertrabecular recesses communicating with the ventricular cavity, with a > twofold thickening of the endocardial noncompacted (NC) layer compared with the epicardial compacted (C) layer of the myocardium (NC/C > 2) [71]. LVNC has been considered a genetic CMP by the AHA, and an unclassified CMP by the ESC. This phenotype is often associated with congenital heart diseases or other CMP, in which genetic analysis follows the patterns of the underlying genetic CMP. There is evidence for wide genetic heterogeneity in LVNC with mutations in sarcomeric, cytoskeletal, Z-line, and mitochondrial proteins. Therefore, it is controversial whether to consider it as a separate entity and it is very rarely encountered in autopsy practice [72, 73]. There is a risk of misnaming a CMP as LVNC when the underlying genetic cause is in fact a HCM, DCM or ACM. We consider LVNC as a phenotypic trait rather than a CMP, that can occur either in isolation or in association with other either congenital heart defects or CMP.

An entity labelled idiopathic LV fibrosis has been put forward as a cause of SCD especially during sport activities [19, 74]. The heart is normal in weight, both ventricles are not dilated and there is no hypertrophy or thinning of the ventricular walls. Fibrosis may be macroscopically visible or not. Microscopically there is significant replacement fibrosis in the LV wall with no myocyte disarray. The distribution of the fibrosis is focal and does not have the patterns seen in other CMP from the histological point of view. This entity, which is also called non ischemic scarring, could be an acquired condition due to healed myocarditis, toxic or drug related but may also be a genetic CMP as familial cases are reported. Thus, the finding of no specific LV scarring should rise the possibility of an inherited CMP in which the phenotype is not well defined.

## The multidisciplinary team approach

The potential genetic origin of heart diseases, including CMP, involves pathologists as part of a multidisciplinary investigation on surviving family members according to AECVP autopsy guidelines and European recommendations established by the Public and Professional Policy Committee of the European Society of Human Genetics (ESHG) [8, 9]. The ESHG recommendations summarize specific procedural, ethical, legal, and practical challenges for post-mortem genetic testing after SCD and indicate how to include postmortem genetic testing in the context of SCD in order to contribute to a better identification of the cause of death, and to a better management of relatives by optimizing screening strategies and treatments of preventable disorders. Their aim is also to stimulate the development of standardized postmortem disclosure policy and procedure at national and international levels for SCD cases and relatives. The role of pathologists in the multidisciplinary team is to perform an autopsy following the AECVP guidelines, to diagnose correctly the case with the help of an expert cardiovascular pathologist if necessary, to store postmortem samples in dedicated biobanks according to legal and ethical guidelines and to help passing on the information either by themselves or via the general practitioner or the cardiologist to the relatives of SCD victims about specific pathway available in their region/country.

A complete autopsy is recommended for SCD victims, as mandatory for deaths under the age of 40 and should be considered for deaths between 40 and 65. These recommendations are also in line with the recent document of the Asia Pacific Heart Rhythm Society and the Heart Rhythm Society [28].

However, adherence to guidelines/recommendations is still suboptimal in many European countries. In up to 40% of cases, autopsies are not performed in subjects less than 50 years who may have died from cardiac disease and only 50% of pathologists declared to follow a standard protocol for autopsy examination, apparently due to lack of expertise and/or training [27, 75].

## Conclusions

While clinical diagnoses of inherited CMP have progressed rapidly with imaging and genetic advances, pathological diagnoses have not progressed at the same pace. There has been as a matter of fact no previous guidelines on these entities. Establishing pathological guidelines is essential as these entities can present with SCD and it is up to the pathologist to establish the diagnosis and raise the possibility of a genetic CMP within the family, thus enabling screening in other family members. The pathologist must also take material for genetic investigation in the deceased (the postmortem genetic testing) when an inherited disease is suspected. The pathologist thus has a pivotal role in the diagnosis and prevention of further deaths within the family and is an essential member of the multidisciplinary team dealing with inherited CMP. Guidelines will change and evolve with advances in knowledge and hopefully uncertain entities will be clarified further.

## Supplementary information

Below is the link to the electronic supplementary material.

Supplementary file1 (DOCX 25 KB)

## Abbreviations

*ACM*: Arrhythmogenic cardiomyopathy
*ACM R*: Arrhythmogenic cardiomyopathy right dominant variant
*ACM L*: Arrhythmogenic cardiomyopathy left dominant variant
*ACM B*: Arrhythmogenic cardiomyopathy biventricular
*AHA*: American Heart Association
*CMP*: Cardiomyopathy
*DCM*: Dilated cardiomyopathy
*ESC*: European Society of Cardiology
*HCM*: Hypertrophic cardiomyopathy
*IVS*: Interventricular septum
*LA*: Left atrium
*LV*: Left ventricle
*LVNC*: Left ventricular non compaction
MOGE (*S*): Morpho-functional (M), organ involvement (O), genetic or familial inheritance pattern (G) and etiological details (E), functional class/status (S)
*RCM*: Restrictive cardiomyopathy
*RA*: Right atrium
*RV*: Right ventricle
*SADS*: Sudden arrhythmic death syndrome
*SCD*: Sudden cardiac death
*SD*: Sudden death
*WHF*: World Heart Federation
