# Supplementary material for: Genetically determined cardiomyopathies at autopsy: the pivotal role of the pathologist in establishing the diagnosis and guiding family screening
Source: Virchows Arch. 2023 Mar 10;482(4):653–69. doi: 10.1007/s00428-023-03523-8 (PMC10067659; doi:10.1007/s00428-023-03523-8)
Supplement: Supplementary file 1 — Supplementary file1 (DOCX 25 KB) [file 428_2023_3523_MOESM1_ESM.docx]

**Supplemental Table 1.** Systematic review of manuscripts on normal heart weight.

| **First author** | **Year** | **Study period** | **Country/po-pulation** | **N.** | **Age range (mean), years** | | **Heart weight mean (SD), g** | | **Heart weight range, g** | | **Predictor** |
| --- | --- | --- | --- | --- | --- | --- | --- | --- | --- | --- | --- |
|  |  |  |  |  | **Men** | **Women** | **Men** | **Women** | **Men** | **Women** |  |
| Arnold | 1899 | 1894 – 1898 | USA, hospital cases | 216 (134 M, 82 W) |  |  | 290 | 253 | 250 – 325 | 225 – 300 |  |
| Smith | 1928 |  | USA, hospital cases | 854 (534 M, 320 W) | 18 – 80 | 18 – 80 | 294 | 250 | 137 – 400 | 110 – 375 | BW |
| Zeek | 1942 | 1924 – 1940 | USA, hospital cases | 926 (523 M, 403 F) | 21 – 69 | 21 – 49 | 312 (52) | 252.9 (46.4) | 200 – 424 | 150 – 374 | BL |
| Reiner | 1959 | 1953 – 1955 | USA | 45 (26 M, 19 w) | 34 – 92 | 21 – 68 | 328 | 244 | 256 – 390 | 198 – 279 |  |
| Hayes | 1966 | 1962 – 1962 | Jamaica | 58 (30 M, 28 W) | 43.5 (14) | 43.5 (16.9) | 294.5 (48.5) | 258.5 (49.5) |  |  |  |
| Dadgar | 1979 | Unknown | India | 138 (116 M, 24 W) | 0 – 78 |  | 236.1 (56.81) | 206.6 (78.61) | 60 – 375 | 25 – 375 | BL |
| Kitzman | 1988 | 1960 – 1982 | USA, hospital cases | 765 (373 M, 392 W) | 20 – 99 | |  |  | 164 – 557 | | BW > BSA > BH |
| Hanzlick | 1990 |  | Men | 201 | 20 – 39 |  | 364 (62) |  |  |  | Age and BW |
| Garby | 1993 | 1972 – 1990 | Danish | 964 (630 M, 334 W) | 45 (17) | 41 (18) | 423 (87) | 320 (67) |  |  |  |
| Ogiu | 1997 | 1985 – 1989 | Japan | 4667 (3023 M, 1644 W) | 0 – 95 | 0 – 93 | 292 – 321 (38 – 51) |  |  |  |  |
| Seo | 2000 | 1994 – 1998 | Korea | 422 (215 M, 207 W) | 1 to 76 | 0 – 77 | 305 | 265 | 280 – 340 | 230 – 280 | BSA > BW > others |
| de la Grandmaison | 2001 | 1987 – 1991 | France, forensic cases | 684 (355 M, 329 W) | 42 (17) | 49 (20) | 365 (71) | 312 (78) | 90 – 630 | 174 – 590 | Age-BW-BMI>BH |
| da Cunha | 2002 | 1986 – 1998 | Brasil | 21 | 44.7 (21.8) | | 329.1 (50.4) | |  |  | BW>BMI |
| Kim | 2009 | 2003 – 2005 | Korea | 526 (369 M, 157 W) | 43.4 (12.54) | 44.6 (14.75) | 346.81 (57.90) | 298.79 (62.59) |  |  |  |
| Sheikhazadi | 2010 | 2007 – 2008 | Tehran | 1222 (914 M, 308 W) | 43.4 (17.8) | 45.2 (22.2) | 359.9 (76.6) | 319.2 (86.2) | 209 – 607 | 199 – 540 | BMI > BH |
| Gaitskell | 2011 | 2003 – 2006 | United Kingdom, hospital cases | 384 (204 M, 180 W) | 14 – 98 | | 380 | 329 | 192 – 672 | 197 – 765 | BSA > BW > others |
| Molina | 2012 | 2005 – 2011 | USA, forensic cases | 232 M | 18 – 35 | 0 | 331 (56.7) |  | 188 – 575 |  |  |
| Vanhebost | 2014 | 2007 – 2011 | Switzerland, forensic cases | 288 (170 M, 118 W) | 37.08 (13.41) | 42.2 (15.7) | 357.1 (53) | 289.8 (63.22) | 260 – 550 | 160 – 455 | BSA > BW > others |
| Wingren | 2015 | 1997 – 2013 | Sweden, forensic cases | 27 645 (20144 M, 7501 W) | Adults | Adults | 386.2 (101–994) | 386.2 (101–994) | 103 – 985 | | BH, BSA and BW in a normal to overweighed population |
| Skurdal | 2016 | 2003 – 2012 | Norway, forensic and medical autopsies | 692 (461 M, 231 W) | 20 – 98 (40.6 M, 45.0 W) | | 395.2 (83.0) | 316.3 (69.0) | 190 – 695 | 190 – 600 | BW, BSA |
| Gholamzadeh | 2017 | 2012 – 2015 | Iran, forensic autopsies | 501 (385 M, 116 F) | 15 – 98 (45.32 M, 44.89 F) | | 356.82 (102.27) | 316.53 (81.99) | 118 – 900 | 119 – 510 | Age > BL |
| Vaibhav | 2022 | 1.5 years | India, forensic cases | 137 (104 M, 33 F) | 15 - 55 | | 310.1 (83.97) | 241.2 (71.42) |  |  |  |
| Westaby | 2022 | 2013 – 2020 | United Kingdom | 1062 (701 M, 361 W) | 18 – 82 | 18 – 100 | 374 (64) | 285 (55) | 191 – 498 | 134 – 399 | Sex > BSA > Age |
| Abbreviations: BL, body length; BH, body height; BW, body weight; BSA, body surface area, BMI, body mass index; M, men; W, women. | | | | | | | | | | | |

**References**

Arnold HD. Weight of the "normal" heart in adults. J Boston Soc Med Sci. 1899 Feb 7;3(6):174-184. PMID: 19971229; PMCID: PMC2359562.

Dadgar SK, Tyagi SP, Singh RP, Hameed S. Factors influencing the normal heart weight--a study of 140 hearts. Jpn Circ J. 1979 Feb;43(2):77-82. doi: 10.1253/jcj.43.77. PMID: 449022.

Da Cunha DF, Cunha SF, Reis MA, Teixeira Vde P. Heart weight and heart weight/body weight coefficient in malnourished adults. Arq Bras Cardiol. 2002 Apr;78(4):382-7. English, Portuguese. doi: 10.1590/s0066-782x2002000400005.

de la Grandmaison GL, Clairand I, Durigon M. Organ weight in 684 adult autopsies: new tables for a Caucasoid population. Forensic Sci Int. 2001 Jun 15;119(2):149-54. doi: 10.1016/s0379-0738(00)00401-1.

Gaitskell K, Perera R, Soilleux EJ. Derivation of new reference tables for human heart weights in light of increasing body mass index. J Clin Pathol. 2011 Apr;64(4):358-62. doi: 10.1136/jcp.2010.084574.

Garby L, Lammert O, Kock KF, Thobo-Carlsen B. Weights of brain, heart, liver, kidneys, and spleen in healthy and apparently healthy adult Danish subjects. Am J Hum Biol. 1993;5(3):291-296. doi: 10.1002/ajhb.1310050307.

Gholamzadeh S, Zarenezhad M, Montazeri M, Zareikordshooli M, Sadeghi G, Malekpour A, Hoseni S, Bahrani M, Hajatmand R. Statistical Analysis of Organ Morphometric Parameters and Weights in South Iranian Adult Autopsies. Medicine (Baltimore). 2017 May;96(21):e6447. doi: 10.1097/MD.0000000000006447.

Hanzlick R, Rydzewski D. Heart weights of white men 20 to 39 years of age. An analysis of 218 autopsy cases. Am J Forensic Med Pathol. 1990 Sep;11(3):202-4. doi: 10.1097/00000433-199009000-00005.

Hayes JA, Lovell HG. Heart weight of Jamaicans. Autopsy study of normal cases and cases of hypertension and chronic lung disease. Circulation. 1966 Mar;33(3):450-4. doi: 10.1161/01.cir.33.3.450.

Kim Y, Kim D, Cho SY, Kim M, Yang KM, Lee H, Han S. Statistical Analysis for Organ Weights in Korean Adult Autopsies. Anatomy & Cell Biology. 2009 42, 219-224.

Kitzman DW, Scholz DG, Hagen PT, Ilstrup DM, Edwards WD. Age-related changes in normal human hearts during the first 10 decades of life. Part II (Maturity): A quantitative anatomic study of 765 specimens from subjects 20 to 99 years old. Mayo Clin Proc. 1988 Feb;63(2):137-46. doi: 10.1016/s0025-6196(12)64946-5.

Molina DK, DiMaio VJ. Normal organ weights in men: part I-the heart. Am J Forensic Med Pathol. 2012 Dec;33(4):362-7. doi: 10.1097/PAF.0b013e31823d298b.

Ogiu N, Nakamura Y, Ijiri I, Hiraiwa K, Ogiu T. A statistical analysis of the internal organ weights of normal Japanese people. Health Phys. 1997 Mar;72(3):368-83. doi: 10.1097/00004032-199703000-00004.

Reiner L, Mazzoleni A, Rodriguez FL, Freudenthal RR. The weight of the human heart. I. Normal cases. AMA Arch Pathol. 1959 Jul;68(1):58-73. PMID: 13660586.

Seo JS, Lee SY, Won KJ, Kim DJ, Sohn DS, Yang KM, Cho SH, Park JD, Lee KH, Kim HD. Relationship between normal heart size and body indices in Korean. J Korean Med Sci. 2000 Dec;15(6):641-6. doi: 10.3346/jkms.2000.15.6.641.

Sheikhazadi A, Sadr SS, Ghadyani MH, Taheri SK, Manouchehri AA, Nazparvar B, Mehrpour O, Ghorbani M. Study of the normal internal organ weights in Tehran's population. J Forensic Leg Med. 2010 Feb;17(2):78-83. doi: 10.1016/j.jflm.2009.07.012.

Skurdal AC, Nordrum IS. A retrospective study of postmortem heart weight in an adult Norwegian population. Cardiovasc Pathol. 2016 Nov-Dec;25(6):461-467. doi: 10.1016/j.carpath.2016.07.003.

Smith HL. The relation of the weight of the heart to the weight of the body and of the weight of the heart to age. Am Heart J. 1928 4 (1):79-93. doi:10.1016/s0002-8703(29)90099-5

Vaibhav V, Meshram R, Shukla PK, Kalonia T, Bhute AR. A Preliminary Study of Organ Weight After Histological Exclusion of Abnormality During Autopsy in the Adult Population of Uttarakhand, India. Cureus. 2022 Jul 19;14(7):e27044. doi: 10.7759/cureus.27044.

Vanhaebost J, Faouzi M, Mangin P, Michaud K. New reference tables and user-friendly Internet application for predicted heart weights. Int J Legal Med. 2014 Jul;128(4):615-20. doi: 10.1007/s00414-013-0958-9.

Westaby JD, Zullo E, Bicalho LM, Anderson RH, Sheppard MN. Effect of sex, age and body measurements on heart weight, atrial, ventricular, valvular and sub-epicardial fat measurements of the normal heart. Cardiovasc Pathol. 2022 Nov 25;63:107508. doi: 10.1016/j.carpath.2022.107508.

Wingren CJ, Ottosson A. Postmortem heart weight modelled using piecewise linear regression in 27,645 medicolegal autopsy cases. Forensic Sci Int. 2015 Jul;252:157-62. doi: 10.1016/j.forsciint.2015.04.036.

Zeek PM. Heart weight. I. The weight of the normal human heart. Arch Pathol 1942 34:820-832
